# Prospective use of genomics in the evaluation of sudden cardiac death: results from a national health service population pathway

**DOI:** 10.1016/j.ebiom.2026.106266

**Published:** 2026-05-13

**Authors:** Cian Wade, Richard Sandford, Frances Elmslie, Mary N. Sheppard, Catherine J. Richards, Bernie Croal, Michael Osborn, Yael Ben-Haim, Carmel E. Raymundo, Louise Hunt, Catherine Mason, Steven Cox, Charlene Roe, Nilesh J. Samani, Sue Hill, Bryan Williams, Elijah R. Behr, NHS and Coronial Service Sudden Unexpected Death programme group, Georgiana Neculau, Laura Vilajuana, Kim Clark, Nina Edwards, Elaine Hobson, Claire Kirby, Katie Frampton, Graham Stuart, Bode Ensam, Nigel Wheeldon, Perry Elliott, William Newman, Harshil Dhutia, Greg Mellor, Jan Till, Judy O'Sullivan, Iain Armstrong, Andrew Leatherland, Jennifer Townsen

**Affiliations:** a Cardiovascular and Genomics Research Institute, City St George's, University of London, UK; b East Genomic Medicine Service Alliance, Cambridge, UK; c South West Thames Centre for Genomics, St George's University Hospitals NHS Foundation Trust, London, UK; d Department of Cellular Pathology, University Hospitals of Leicester NHS Trust; e Royal College of Pathologists, London, UK; f North West London Pathology, Imperial College Healthcare NHS Trust, London, UK; g South East Genomic Medicine Service Alliance, London, UK; h HM Coroner's Service for Birmingham and Solihull, Birmingham, UK; i HM Coroner's Service for Leicester City & South Leicestershire, Leicester, UK; j Cardiac Risk in the Young, Surrey, UK; k NHS England, Wellington House, London, UK; l Department of Cardiovascular Sciences, University of Leicester, Leicester, UK; m British Heart Foundation, Greater London House, London, UK

**Keywords:** Inherited cardiac condition, Sudden cardiac death, Genomics, Cardiac pathology, Population health, Pathway transformation

## Abstract

**Background:**

Sudden cardiac death (SCD) is a major cause of premature and potentially avoidable mortality. Determining whether a SCD was caused by an inherited cardiac condition (ICC) enables identification and appropriate clinical management of at-risk relatives. We report findings from the NHS and Coronial Service Sudden Unexpected Death programme, which established a national pathway aimed at reducing population risk of SCD in England.

**Methods:**

Cases of SCD were included where a coroner's autopsy raised suspicion of an ICC (ages 1–60) or was unable to determine a cause of death (sudden arrhythmic death syndrome [SADS]) (ages 1–40). Decedent tissue was retained for post-mortem genetic testing, and their relatives were signposted to a new ICC pathway. Where a decedent harboured a pathogenic (P) or likely pathogenic (LP) genetic variant associated with an ICC, relatives were offered predictive genetic testing for that variant. A negative genetic result enabled appropriate discharge, while a positive result triggered clinical evaluation for cardiac disease phenotype. Where no ICC associated P or LP variants were identified in decedents, their relatives underwent clinical evaluation.

**Findings:**

Of 107 SCD cases, the most common findings were SADS (35%) and arrhythmogenic cardiomyopathy (15%), while 17% of those tested were found to have P or LP variants. Three-hundred-and-seven relatives subsequently entered the ICC clinic for further evaluation. Diagnostic yield for relatives of decedents with P or LP variants was 47%. Of the 235 relatives who completed ICC evaluation, 28% were provided with a new genetic and/or clinical diagnosis and subsequently managed appropriately. One notable family cluster (n = 24) included 13 individuals (57%) with the *KCNH2*:c2775dup variant causative of long QT syndrome.

**Interpretation:**

Integration of coronial, pathology, genomic, and cardiac services in a prospective population pathway enabled efficient capture and use of genetic and clinical information to reduce population risk of SCD.

**Funding:**

10.13039/501100000274British Heart Foundation, Cardiac Risk in the Young, NHS England, 10.13039/501100000272NIHR.

**Research in context**

**Evidence before this study**

Sudden cardiac death (SCD) is a devastating and potentially avoidable source of mortality that may be caused by an inherited cardiac condition (ICC). Autopsy with expert examination and post-mortem genetic testing of the deceased, as well as genetic and/or clinical evaluation of their at-risk relatives are essential for identifying and reducing population risk of SCD. National prospective pathways that deliver all these components have not been studied to understand their real-world effectiveness.

**Added value of this study**

The NHS and Coronial Service Sudden Unexpected Death programme established a coordinated pilot pathway across England to optimise and standardise the evaluation of decedents aged 1–60 and their relatives who experienced SCD due to a suspected ICC. This national programme was unique in its prospective integration of coronial, pathology, genomic, and cardiac services to inform targeted management of relatives. We describe the process and yield of the genetic and clinical evaluation of 107 decedent SCD cases and their 307 relatives who entered the ICC pathway. Our programme provided 28% of relatives with a new genetic and/or clinical diagnosis that informed onward appropriate management of their cardiac risk and enabled the safe discharge of a further 69%. We demonstrate how prospective genetic testing can efficiently stratify the population at-risk of SCD according to those requiring detailed further clinical evaluation and those who can be reassured and discharged.

**Implications of all the available evidence**

Our findings suggest that a pathway-based transformation that integrates prospective genetic and clinical evaluation may contribute to reducing avoidable deaths by identifying ICCs within families. This offers a genomics-centred model for health systems to manage population risk of SCD.

## Introduction

Sudden unexpected death (SUD) with a cardiac cause, sudden cardiac death (SCD), is a major global health problem, with an estimated incidence varying from 1.3 to 159 per 100,000 persons per annum depending on the age range considered.^1^, ^2^, ^3^ In older populations, SCD is associated with other causes such as coronary artery disease. The annual incidence of SCD in 1-35-year-olds is estimated at 1.68 per 100,000, with lower rates in children and higher rates in young adults.^3^^,^^4^ Inherited cardiac conditions (ICCs), including cardiomyopathies, inherited arrhythmia syndromes, and aortopathies, are the predominant causes, and may account for up to three quarters of cases.^5^

SCD can be the first presentation of a previously undiagnosed ICC in a family; therefore, establishing a diagnosis permits identification of other relatives who may be at risk.^5^, ^6^, ^7^ The optimal pathway requires three components for the comprehensive personalised management of families: initial autopsy examination with specialist cardiac pathology and toxicological studies^8^; post-mortem genetic testing if an ICC is suspected or the cause is unexplained, known as the sudden arrhythmic death syndrome (SADS)^9^; and familial genetic and clinical evaluation guided by the results of post-mortem genetic testing.^5^ Each of these components are typically separately funded and governed, creating significant hurdles to an integrated service. Indeed, patient pathways capable of providing the appropriate evaluation of these families are highly variable across England and internationally.^10^ Expert pathology has been largely contingent on charitable funding by Cardiac Risk in the Young (CRY) and there is no established working pathway between the coronial service and the NHS.^8^^,^^11^

To address the variation in access to ICC services and ensure optimal delivery of care, the NHS and Coronial Service Sudden Unexpected Death (NHS-C-SUD) programme (referred to as the “programme”) was established to enhance and standardise the patient pathway for families following a SCD in individuals aged 1–60 years.^12^ The programme's uniqueness was the prospective integration of coronial, pathology, genomic and cardiac services into an optimised pathway that enabled timely post-mortem genetic testing and subsequent appropriate genetic and clinical evaluation of relatives to identify and manage population risk of SCD. We performed an analysis of this programme, focussing on the genetic and clinical outcomes of decedents and their relatives referred into this new ICC pathway.

## Methods

### Establishing the programme pilot sites

The NHS-C-SUD programme was launched in 2021 with programme management by the Healthcare Innovation team within the British Heart Foundation (BHF). The first pilot sites initiated set-up in February 2022, with most continuing until March 2024. Seven sites were established, one in each of the seven Genomic Medicine Service Alliance (GMSA) regions within NHS England. Each site consisted of at least one HM Coroner's district, the geographically co-located ICC clinical service, and its associated Genomic Laboratory Hub (GLH, genetic testing service). Sites were based in Bristol, South London, North London, Birmingham, Leicester, Manchester, and Sheffield. The National Oversight board included senior leadership representing NHS England, the BHF, the Chief Coroner, the Royal College of Pathologists, and Cardiac Risk in the Young (CRY), as well as Coroner, Clinical and Scientific leads. The National Steering Committee was established to report to the Oversight Board and provide support to pilot sites. This committee consisted of coroners, CRY representatives, and national and pilot site leads with clinical, scientific and pathological expertise in ICCs and genomics. A Data Protection Impact Assessment (DPIA) was performed to minimise any data protection risks associated with the pseudo-anonymised patient data collected during the programme.

**Fig. 1:**
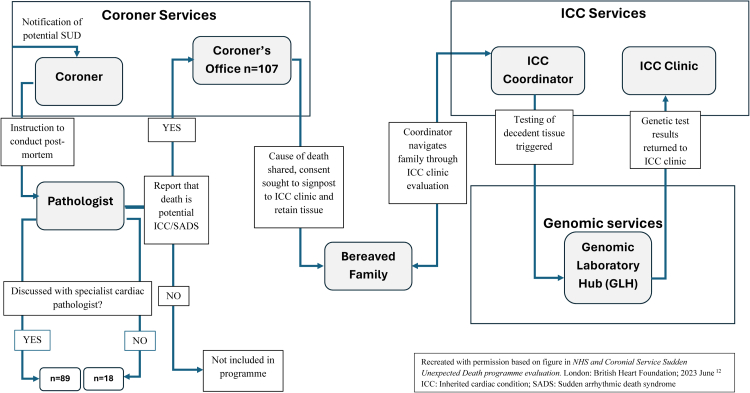
**Outline of the NHS-C-SUD pathway**.

A new standardised pathway was established across each pilot site, supported by training sessions and educational materials for coronial staff, ICC teams and relatives (Fig. 1). The pathway aimed to identify appropriate SCD decedents, implement post-mortem genetic testing, and prospectively genetically and/or clinically evaluate relatives of decedents.

### Decedent inclusion

All SUD cases aged 1–60 years that were reported to HM Coroners in the participating pilot sites were considered for inclusion and underwent autopsy by the coroner's pathologist with expert cardiac pathological assessment as required. If an ICC was suspected, or the deaths remained unexplained despite a full coronial and expert cardiac autopsy examination and toxicological testing, designated as SADS, then the cases were included in the pilot and suitable tissue retained for DNA extraction and future genetic testing (included causes of death in Supplementary Table 1). Retained tissue included splenic samples and were stored either frozen at −80 °C or in RNAlater solution. Toxicological testing was performed in every case where the death remained unexplained after autopsy examination, or where a toxicological contributor to death was specifically suspected. Toxicological testing reported the presence of medications and drugs. However, a case was excluded only when the Coroner's conclusions, informed by the pathologist and toxicologist's advice, indicated that the cause of death was due to overdose or toxicity.

### Relative inclusion and genetic testing

Where a decedent met the inclusion criteria, their relatives were signposted by the coroner's officer to regional ICC services, who would then initiate contact. An ICC coordinator (typically a specialist cardiac genetic nurse) would then interview relatives, take consent for post-mortem genetic testing of retained tissue, instigate testing through the local GLH and map family trees to identify all living 1st degree relatives eligible for further evaluation. Where the autopsy revealed macroscopic and microscopic evidence of an ICC, post-mortem genetic testing was targeted with a clinical panel for that condition (Supplementary Table 2). Two different sequencing methodologies were employed by GLHs. One was clinical whole exome sequencing (WES) and the other candidate gene panel sequencing. Genes were selected for testing in virtual panels according to the underlying phenotype, or the absence of phenotype, at autopsy. SADS cases aged 1–40 underwent testing with the current wide clinical ‘molecular autopsy’ panel. Cases where autopsy findings were borderline or showed overlap between ICCs were designated as *Other* and were considered for post-mortem genetic testing on a case-by-case basis. Non-1st degree relatives were also included according to clinical need if they were symptomatic or likely to be an obligate carrier.

Genetic test results were categorised according to Human Genome Variation Society (HGVS)^13^ nomenclature. American College of Medical Genetics (ACMG) guidelines were used by GLHs to identify genetic variants that were pathogenic (P, class 5) or likely pathogenic (LP, class 4).^14^ Where the decedent was found to have a P or LP variant associated with an ICC, their relatives were offered cascade predictive genetic testing for that variant by Sanger sequencing at each GLH (Supplementary Figure 1). A relative who was subsequently found to be genotype negative was discharged without further clinical evaluation, unless required to exclude polygenic disease. Genotype positive relatives were then clinically evaluated by consultant cardiologists (adult and paediatric) with a special interest in ICCs. This included a review of medical and family history, clinical examination, and cardiac investigations deemed appropriate to the case, such as ECGs, transthoracic echocardiogram, exercise testing, ajmaline challenge and cardiac MRI.^5^^,^^15^ Where the decedent tested negative for an ICC associated genetic variant, relatives were offered clinical evaluation. Variants of uncertain significance (VUS) were not reported. A standardised database was developed for ICC coordinators across each pilot site to log decedent data, relative demographics, clinical history, and genetic & clinical evaluation outcomes.

### Analysis of genetic & clinical evaluation outcomes

Genetic and clinical findings of participants continued to be collected until April 2025. The proportion of positive genetic tests were calculated to determine the programme's genetic testing yield. Relatives who underwent clinical evaluation were designated as phenotype positive or negative, dependent on whether specific cardiac diagnoses were made. Subpopulations of interest included relatives where the decedent was genotype positive or negative.

### Ethics

This study was conducted as a service evaluation of a clinical pathway using anonymised data. Formal NHS Research Ethics Committee review was not required.

### Statistical analysis

The results of the programme were mainly descriptive, with limited comparative statistical analysis performed in Stata.^16^

### Role of funders

Funders had no role in analysis, interpretation or article writing. No funders had any role in the study design, data collection, data analyses, interpretation, or writing of the report.

## Results

### Decedent autopsy and genetic findings

A total of 107 decedents were identified by HM Coroners’ pathologists who died between March 2021 and January 2024 and were included in the programme. Median age of death was 35 years (IQR 27.5–45.5) with 67% of cases occurring in males and 33% in females (Supplementary Table 3). The most common circumstances of death were at rest (45%), during sleep (16%), and while exercising (14%). Sixty-five percent of decedents were reported as being from White ethnic groups, 9% from Black groups, and 7% from Asian groups, with the remainder not reported.

The most common single cause of death among decedents was SADS with 37 cases (35%) (Table 1). Eight cases of SADS were included in decedents >40 years old due to additional family history of sudden death. Cardiomyopathies composed the next largest group, including 16 cases (15%) of arrhythmogenic cardiomyopathy (ACM), 9 cases (8%) of dilated cardiomyopathy (DCM), and 7 cases (6%) of hypertrophic cardiomyopathy (HCM). Other causes included thoracic aortic aneurysm and its complications (9 cases), unexplained cardiac hypertrophy (7 cases), unexplained cardiac scarring/fibrosis (4 cases), severe mitral valve disease (4 cases), and other causes (12 cases).

**Table 1:** Decedent causes of death and genetic status.

Cause of death as documented on the MCCD	# Count (%)	#P/LP variant positive (%)^a^
1a. Sudden arrhythmic/adult death syndrome (SADS)	37 (35%)	3 (10%)
1b. Unascertained: Equivocal findings	2 (2%)	0 (0%)
2a. Hypertrophic cardiomyopathy (HCM)	7 (7%)	3 (43%)
2b. Dilated cardiomyopathy (DCM)	9 (8%)	0 (0%)
2c. Arrhythmogenic cardiomyopathy (ACM)/arrhythmogenic right ventricular cardiomyopathy	16 (15%)	4 (33%)
2d. Unexplained cardiac hypertrophy	7 (6%)	1 (20%)
2e. Unexplained cardiac scarring/fibrosis	4 (4%)	1 (25%)
3. Severe mitral valve prolapse with myxomatous degenerative valvular disease	4 (4%)	1 (25%)
4. Thoracic aortic aneurysm ± dissection/rupture	9 (8%)	2 (22%)
5. Other	12 (11%)	0 (0%)
Total	107	15 (17%)

MCCD: Medical certificate of cause of death; P: Pathogenic; LP: Likely pathogenic.

a Proportion of positive cases among those who completed genetic testing within each cause of death group (total n = 89).

Based on an estimated SCD incidence range of 1.68 per 100,000,^4^ 96 cases of SCD would have been expected across the 7 pilot sites during a 12-month period (Supplementary Table 4). During a selected 12-month period when all pilot sites were fully operational, 63 SCD decedents with a likely genetic basis were included.

Targeted clinical panel testing or wide clinical molecular autopsy panel were positive in 15 of the 89 decedents who had completed their testing by the end of the programme (17%). Of these 10/15 (66%) were P and 5/15 (33%) were LP (Table 1). The most frequently identified genes with relevant variants were *DSP* (3 cases of ACM) and *MYBPC3* (2 cases of HCM and 1 case of severe mitral valve prolapse) (Table 2). The causes of death with the highest proportion of P/LP variants among those tested were HCM with 3 out of 7 (43%) cases positive and ACM with 4 out of 12 (33%) cases positive.

**Table 2:** Decedent genetic variants and associated cause of death.

Gene	Nucleotide change	Amino acid change	ACMG class	Associated cause of death
*KCNH2*	c.2775dup	p.(Pro926AlafsTer14)	5	SADS
*RYR2*	c.1259G>A	p.(Arg420Gln)	5	SADS
*SCN5A*	c.5000T>A	p.(Val1667Asp)	4	SADS
*MYBPC3*	c.2096delC	p.(Pro699GlnfsTer55)	5	HCM
*MYBPC3*	c.1504C>T	p.(Arg502Trp)	5	HCM
*LAMP2*	chrX:120431322-120469169	n/a	5	HCM
*DSP*	c.7773_7776del	p.(Ser2591ArgfsTer11)	4	ACM/ARVC
*DSP*	c.5232dup	p.(Lys1745)	5	ACM/ARVC
*DSP*^a^	c.3805C>T	p.(Arg1269^b^)	5	ACM/ARVC
*JUP*^a^	c.2039G>A	p.(Trp680^b^)	5	ACM/ARVC
*FLNC*	c.4333_4340delins20	p.(Lys1445_Lys1447delinsProGlyGlnTer)	5	ACM/ARVC
*MYH7*	c.732+1G>A	n/a	5	Unexplained cardiac hypertrophy
*KCNE1*	c.226G>A	p.(Asp76Asn)	4	Unexplained cardiac scarring/fibrosis
*MYBPC3*	c.1224-19G>A	n/a	5	Severe MV prolapse with myxomatous degenerative valvular disease
*COL3A1*	c.528+5G>A	n/a	4	Thoracic aortic aneurysm
*FBN1*	c.1426T>A	p.(Cys476Ser)	4	Thoracic aortic aneurysm

n/a Refers to variants where the corresponding amino acid impact is unpredictable (e.g. splice variants).

a Note that these two variants co-existed in a single deceased case of ACM/ARVC.

b After amino acid change denotes nonsense variant.

### Genetic & phenotypic yields for relatives

**Fig. 2:**
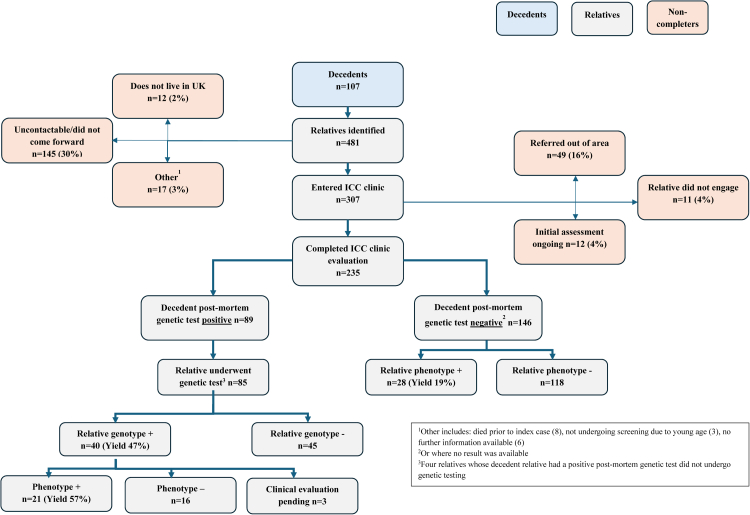
**Flow of relatives through the pathway**.

Of the 107 decedents, a total of 481 relatives were initially identified with 307 of these successfully contacted and agreeing to enter the ICC clinic (Fig. 2). At least one relative entered the ICC from 90 (84%) of the decedents. A total of 235 (77%) of these relatives completed their ICC clinic evaluation (mean of 2.6 and median of 3 per decedent where at least one relative entered the ICC). Reasons for non-completion included being referred out of area (49), not engaging with the ICC clinic (11), and initial assessment still ongoing at the end of the programme (12). Where recorded, the average duration between decedent date of death and date of clinical evaluation of the relative in the ICC clinic was 225 days.

Eighty-five relatives of the 15 decedents who had positive post-mortem genetic testing results underwent predictive genetic testing. Forty (47% genetic yield) of these relatives were identified as having a P/LP variant. Of these 40 genotype positive cases, 37 had completed clinical evaluation by the end of the programme and 21 of these had cardiac disease (57% phenotypic yield). The largest single-family cluster was 13 individuals out of 24 relatives identified as having the *KCNH2*-c.2775dup variant (decedent case identifier 7001, Supplementary Table 5). Five of these 13 were diagnosed with definite Long QT syndrome, while 7 others had supportive symptoms. Forty-five relatives with negative predictive genetic testing were discharged without further evaluation.

One-hundred-and-forty-six relatives of the 92 decedents with negative post-mortem genetic testing results or with no result available completed clinical evaluation only. Twenty-eight out of 146 (19% phenotypic yield) were found to have a cardiac disease phenotype, with the single most identified pathology being DCM (n = 4). Across all relatives, the programme achieved an overall genetic yield of 47% (40 of 85 tested) and phenotypic yield of 27% (49 out of 183 tested).

Overall, 28% of relatives who completed their ICC clinic evaluation were found to have a new genetic and/or clinical diagnosis requiring ongoing management in the ICC clinic (Table 3). 69% had negative genetic or phenotypic findings that enabled safe discharge after their ICC evaluation. 3% were still awaiting an outcome from the ICC clinic at the end of the programme's analysis period. A detailed list of genetic and clinical findings by decedent case identifier can be found in Supplementary Table 5.

**Table 3:** Relative outcomes by group.

Overall outcome group	Cases	Proportion	Programme action
G+ P+	21	9%	Ongoing management in the ICC clinic
G+ P−	16	7%	
P+ no G test performed	28	12%	
G− no P test needed^a^	45	19%	Discharged^b^
P− no G test performed	118	50%	
G/P still pending	7	3%	Await outcome

G: Genotype; P: Phenotype.

a In the absence of likely polygenic disease.

b Children received ongoing follow-up in the ICC clinic in case of age-related disease penetrance.

## Discussion

We report the effectiveness of a national pathway that integrates coronial, genomic, and cardiac services to reduce risk of SCD in the population. Previous studies have primarily been retrospective research analyses and focused on the clinical evaluation of relatives.^17^^,^^18^ Where genetic testing has been performed, this has occurred as part of an isolated research study and/or after initial clinical evaluation of relatives, rather than as part of a prospective population pathway that could sustainably deliver this service.^19^^,^^20^

There are several indicators that the programme was effective in identifying decedents and evaluating their relatives at risk of SCD. Applying estimates of annual SCD incidence to the 7 pilot sites indicated the programme included a slightly lower number of cases over a select 12-month period. However, this was expected as it reflected that the programme targeted only the subset of SCD cases where an ICC was suspected. We therefore anticipate that a majority of incident SCD cases with a suspected genetic basis would be identified and recruited to the pathway if rolled out nationally. Seventeen percent of tested decedent cases were identified as having a P or LP variant associated with an ICC. This is consistent with published estimates from other SCD cohorts, indicating that the programme population is representative of that likely to be encountered in clinical practice.^20^, ^21^, ^22^

Of relatives undergoing cascade predictive genetic testing, 47% were identified as having a P/LP variant. This high yield reflects the judicious use of genetic testing in relatives of genotype positive decedents only. Following clinical assessment, the overall phenotypic yield of the programme was 27%. Strikingly, this was achieved in a real-world clinical setting with a comparable yield to stand-alone research studies.^5^^,^^23^^,^^24^ The programme demonstrated a higher phenotypic yield of 57% among relatives who were genotype positive. Thus, nearly half of relatives with risk from ostensibly monogenic disease were identifiable only through genetic, rather than clinical, evaluation. Furthermore, 53% of relatives with a genotype positive decedent had a negative genetic test and could be discharged provided there were no suspicions of polygenic disease, such as the Brugada syndrome.^25^ The utility of this approach is appreciated in family cluster 7001 where the decedent's cause of death was designated as SADS (Supplementary Table 5). Identification of the *KCNH2*-c.2775dup variant in the decedent triggered cascade genetic testing in the rest of the family. Of the 24 who completed testing, 11 were genotype negative and could be safely discharged while 13 harboured the variant and underwent further clinical evaluation. Twelve had clinical evidence of Long QT syndrome, 5 of whom fulfilled definite clinical diagnostic criteria.^5^ Without expeditious molecular autopsy in the decedent and efficient evaluation of the family enabled by this programme, these relatives may have not received these diagnoses and been appropriately managed to reduce their risk of SCD.

Our findings highlight the value of prospective genetic testing as a population risk stratification tool. Targeted genetic testing can identify relatives with variably penetrant disease who may otherwise be missed and those that can be safely discharged without resource-intensive and sometimes distressing clinical testing. This programme is therefore a compelling example of fulfilling government ambitions of harnessing genomics to improve population health.^26^

The programme achieved a high evaluation completion rate (77%) for relatives entering the ICC clinic, with only 4% not completing due to disengagement. Seventy percent of relatives of genotype positive decedents entered the ICC clinic and 97% of those completed their predictive genetic testing, where eligible. These high completion rates indicate that the pathway successfully engaged relatives through what is often a complex and emotional patient journey.^27^ The ICC coordinator who liaised between the bereaved family, the ICC clinic, and the GLH, was a key part of improving the family's experience. Qualitative interviews outside the scope of this analysis reported that the coordinator as a single point of contact significantly improved family engagement and overall efficiency of the pathway.^12^

Building on prior work to increase autopsy examination rates for suspected SCD, the pathway improved on several key issues frequently encountered by families.^8^ Previously, Coroners' Officers would not routinely obtain consent from families for the retention of tissue from the decedent, representing a lost opportunity for many families.^28^ Under the new pathway, pathologists prompt Coroners’ Officers to seek this consent from the family in every appropriate case to increase SCD diagnosis rates. Even when tissue was retained there was often inconsistency in the types of samples taken, including not routinely storing DNA rich tissues, and inappropriate labelling and storage, resulting in lower DNA yields. In line with the new protocol, tissue samples are handled appropriately with an end-to-end process for tracking samples, which may have improved diagnostic yield and speed in this programme.

Notwithstanding recent progress, historically there has been significant variation in access to a specialist cardiac pathology service examination leading to delays and potentially misdiagnoses of SCD.^29^^,^^30^ In this programme, pathologists were supported with access to specialist cardiac pathology, mostly via the CRY Centre for Cardiac Pathology, to assist in case identification. Furthermore, relatives would previously be advised to obtain a referral to the ICC clinic via their GP. Anecdotal evidence suggests that this resulted in fewer referrals to the ICC clinic and missed opportunities to identify relatives at risk of SCD.^12^ Under the new pathway, the family are flagged by the Coroner's Officer to the NHS ICC service and the ICC coordinator initiates the family assessment. Consequently, the burden for ensuring appropriate evaluation is removed from the family and a greater number of individuals may ultimately enter the ICC clinic.

Several challenges exist in enabling a sustainable national roll-out of this programme. Foremost are the additional resources required within ICC and cardiac pathology services to enable timely evaluation of decedent cases. Cardiac pathology is not a formally recognised subspeciality and, in the UK, has relied upon a combination of charitable and research funding, ad hoc local authority support, and professional good will to take on additional work. Some logistical issues were also encountered. These included delays in transporting decedent tissue to GLHs and low awareness of the new pathway within some coronial and clinical services, particularly where staff turnover is high. We also noted a significant bimodal distribution for the proportion of relatives completing their ICC evaluation where at least one relative entered the ICC clinic. While 57% of families had a 100% completion rate, 26% had a 0% completion rate, indicating the critical importance of early and ongoing family engagement throughout ICC clinic evaluation. Although not formally evaluated, anecdotal feedback cited several barriers to family engagement. This included language barriers where the objectives of onward evaluation of relatives were insufficiently communicated to certain families. While we did provide a range of translated materials, future rollout should ensure a broader range of linguistically and culturally appropriate materials to maximise engagement. Additionally, some families reported being too traumatised after the death of their family member to engage with ICC services. In these cases, contact details were provided to the families so that they could re-refer themselves at a future date. Finally, 49 relatives did not complete their ICC evaluation due to being referred out of area. While many may have ultimately completed cardiac evaluation in non-pilot ICC centres, national coordination is required to ensure that none are lost within the system and genetic data is shared appropriately.

Limitations of this analysis include the absence of a comparator arm. While genetic and phenotypic yields were higher than those reported in other studies, we cannot quantify the iterative value of this programme. A limited cost benefit assessment has been performed to support the case for long-term centralised funding.^12^ The primary additional costs included the ICC coordinators and additional capacity for specialist cardiac pathology. Anecdotal reports from coroners participating in the programme indicated that the pathway created minimal additional workload and was easily incorporated into existing workflows. While programme costs are likely to be negligible compared to the economic and social costs of SCD in young people, further work should include a formal cost benefit analysis incorporating the impact of lives saved over their life course.

Another limitation is the lack of full access to data relating to the coronial evaluation of decedents. The contribution of positive toxicological results toward the overall cause of death can be challenging to interpret. We relied upon the expert judgement of the pathologist, specialist cardiac pathologist, and toxicologist. However, given that the objective of this programme was to evaluate the real-world outputs of a new clinical pathway, we would suggest that this approach is most instructive for its likely performance during future activity.

In conclusion, this programme established a prospective pathway that enabled streamlined evaluation of families where an ICC was suspected as the cause of a relative's death. Integration of coronial, pathology, genomic and cardiac services generated genetic and phenotypic insights that directly informed onward clinical management of relatives. This provides a model for how a genomics and pathway-based transformation can enable health systems to address reduction of risk of SCD in their population.

## Contributors

RS, FE, MNS, CJR, MO, SC, NJS, SH, and ERB conceptualised the design and evaluation of the NHS-C-SUD programme. NJS, SH, BW, and ERB provided overall supervision of the programme and evaluation. MNS, BC, CJR, LH, CM, provided leadership for the autopsy evaluation of decedents. CR coordinated NHS England's role in the programme. CW, Y B-H, and CER coordinated data acquisition, accessed and verified the validity of the data related to decedents and relatives. CW performed the data analysis. CW and ERB wrote the original draft. All authors read and approved the final version of the manuscript. The NHS-C-SUD programme group members contributed to the design, implementation, and ongoing management of the ICC pathway detailed in this manuscript.

## Data sharing statement

The individual participant data analysed in the Article were collected as part of the NHS-C-SUD programme. Data was anonymised, securely stored, and transferred for the purposes of this analysis. Any requests for access to this data should be directed toward Professor E R Behr and will be subject to the approval of data owners and applicable data sharing agreements.

## Declaration of interests

CW's role was funded by the National Institute for Health and Care Research. MNS's role is part funded by CRY; SC is the Chief Executive Officer of CRY; CR is employed by NHS England; NJS was previously Medical Director of the BHF, SH is the Chief Scientific Officer at NHS England; BW is the Chief Scientific and Medical Officer at BHF; ERB receives BHF and NHS England research grant funding.

## Appendix A Supplementary data

Supplementary Tables and Figure

Collab Authors

## References

[1] Stiles M.K., Wilde A.A.M., Abrams D.J. (2021). 2020 APHRS/HRS expert consensus statement on the investigation of decedents with sudden unexplained death and patients with sudden cardiac arrest, and of their families. J Arrhythm.

[2] Wong C.X., Brown A., Lau D.H. (2019). Epidemiology of sudden cardiac death: global and regional perspectives. Heart Lung Circ.

[3] Lynge T.H., Risgaard B., Banner J. (2021). Nationwide burden of sudden cardiac death: a study of 54,028 deaths in Denmark. Heart Rhythm.

[4] Bhatia R.T., Yeo T.J., MacLachlan H. (2024). Insights into the aetiology and temporal trends in cardiac mortality in the young: a 21-year review of national and registry data in the United Kingdom. Eur Heart J.

[5] Zeppenfeld K., Tfelt-Hansen J., de Riva M. (2022). 2022 ESC Guidelines for the management of patients with ventricular arrhythmias and the prevention of sudden cardiac death. Eur Heart J.

[6] Behr E.R., Wood D.A., Wright M. (2003). Cardiological assessment of first-degree relatives in sudden arrhythmic death syndrome. Lancet.

[7] Behr E.R., Dalageorgou C., Christiansen M. (2008). Sudden arrhythmic death syndrome: familial evaluation identifies inheritable heart disease in the majority of families. Eur Heart J.

[8] Sheppard M.N., Westaby J., Zullo E. (2023). Sudden arrhythmic death and cardiomyopathy are important causes of sudden cardiac death in the UK: results from a national coronial autopsy database. Histopathology.

[9] Gray B., Behr E.R., Papatheodorou E. (2024). Influence of age and sex on the diagnostic yield of inherited cardiac conditions in sudden arrhythmic death syndrome decedents. Eur J Prev Cardiol.

[10] Behr E.R., Scrocco C., Wilde A. (2022). Investigation on sudden unexpected death in the young (SUDY) in Europe: results of the European Heart Rhythm Association Survey. Europace.

[11] Bowker T.J., Wood D.A., Davies M.J. (1995). Sudden unexpected cardiac death: methods and results of a national pilot survey. Int J Cardiol.

[12] British Heart Foundation (2023). http://www.bhf.org.uk/-/media/files/for-professionals/healthcare-professionals/nhs-c-sud-interim-evaluation-report-final.

[13] den Dunnen J.T., Dalgleish R., Maglott D.R. (2016). HGVS recommendations for the description of sequence variants: 2016 update. Hum Mutat.

[14] Richards S., Aziz N., Bale S. (2015). Standards and guidelines for the interpretation of sequence variants: a joint consensus recommendation of the American College of Medical Genetics and Genomics and the Association for Molecular Pathology. Genet Med.

[15] Behr E.R., Winkel B.G., Ensam B. (2025). The diagnostic role of pharmacological provocation testing in cardiac electrophysiology: a clinical consensus statement of the European Heart Rhythm Association and the European Association of Percutaneous Cardiovascular Interventions (EAPCI) of the ESC, the ESC Working Group on Cardiovascular Pharmacotherapy, the Association of European Paediatric and Congenital Cardiology (AEPC), the Paediatric & Congenital Electrophysiology Society (PACES), the Heart Rhythm Society (HRS), the Asia Pacific Heart Rhythm Society (APHRS), and the Latin American Heart Rhythm Society (LAHRS). Europace.

[16] StataCorp L.L.C. (2025).

[17] Bagnall R.D., Weintraub R.G., Ingles J. (2016). A prospective study of sudden cardiac death among children and young adults. N Engl J Med.

[18] Webster G., Olson R., Schoppen Z.J. (2019). Cardiac evaluation of children with a family history of sudden death. J Am Coll Cardiol.

[19] Quenin P., Kyndt F., Mabo P. (2017). Clinical yield of familial screening after sudden death in young subjects: the French experience. Circ Arrhythm Electrophysiol.

[20] Ripoll-Vera T., Pérez Luengo C., Borondo Alcázar J.C. (2021). Sudden cardiac death in persons aged 50 years or younger: diagnostic yield of a regional molecular autopsy program using massive sequencing. Rev Esp Cardiol (Engl Ed).

[21] Tester D.J., Ackerman M.J. (2007). Postmortem long QT syndrome genetic testing for sudden unexplained death in the young. J Am Coll Cardiol.

[22] Chmielewski P., Świerczewski M., Foss-Nieradko B. (2024). Clinical and genetic yield of familial screening after a sudden unexplained death at a young age. Kardiol Pol.

[23] Kumar S., Peters S., Thompson T. (2013). Familial cardiological and targeted genetic evaluation: low yield in sudden unexplained death and high yield in unexplained cardiac arrest syndromes. Heart Rhythm.

[24] Siskind T., Williams N., Sebastin M. (2022). Genetic screening of relatives of decedents experiencing sudden unexpected death: medical examiner's office referrals to a multi-disciplinary cardiogenetics program. J Community Genet.

[25] Papadakis M., Papatheodorou E., Mellor G. (2018). The diagnostic yield of Brugada syndrome after sudden death with normal autopsy. J Am Coll Cardiol.

[26] Sheppard M.N. (2022). Sudden unexpected death: a national programme which will establish genetic testing and cardiological screening of families in the UK. Pathologie (Heidelb).

[27] Zhang D., Liu Y., Chen N., Li Y., Li X. (2024). Experiences and needs of family members following sudden cardiac death: a meta-synthesis. Int J Nurs Stud.

[28] Fellmann F., van El C.G., Charron P. (2019). European recommendations integrating genetic testing into multidisciplinary management of sudden cardiac death. Eur J Hum Genet.

[29] Cox S. (2025). Cardiac Risk in the Young: 30 years of supporting families and preventing young sudden cardiac deaths. Br J Cardiol.

[30] de Noronha S.V., Behr E.R., Papadakis M. (2014). The importance of specialist cardiac histopathological examination in the investigation of young sudden cardiac deaths. Europace.

