# Supplementary material for: Prospective use of genomics in the evaluation of sudden cardiac death: results from a national health service population pathway
Source: eBioMedicine. 2026 May 13;128:106266. doi: 10.1016/j.ebiom.2026.106266 (PMC13261715; doi:10.1016/j.ebiom.2026.106266)
Supplement: Supplementary Tables and Figure [file mmc1.docx]

**Supplementary Materials**

**Supplementary table 1: Eligibility criteria for decedent inclusion in programme**

| **Cause of death** | **Age range for eligibility for post-mortem genetic testing** |
| --- | --- |
| 1a. Sudden arrhythmic death syndrome (SADS) | 1-40* |
| 1b. Unascertained: Equivocal findings | 1-60 |
| 2a. Hypertrophic cardiomyopathy (HCM) | 1-60 |
| 2b. Dilated cardiomyopathy (DCM) | 1-60 |
| 2c. Arrhythmogenic cardiomyopathy (ACM)/arrhythmogenic right ventricular cardiomyopathy | 1-60 |
| 2d. Unexplained cardiac hypertrophy | 1-60 |
| 2e. Unexplained cardiac scarring/fibrosis | 1-60 |
| 3. Severe mitral valve prolapse with myxomatous degenerative valvular disease | 1-40* |
| 4. Thoracic aortic aneurysm +/- dissection / rupture | 1-40* |
| 5. Other | 1-60 |

*Decedents >40 years old considered if there was additional family history of sudden death

**Supplementary table 2: Key phenotypes with corresponding Genomic Test Directory Reference Number**

| **Genomic test directory reference** | **Phenotype** |
| --- | --- |
| R125 | Thoracic aortic aneurysm or dissection |
| R138 | SADS molecular autopsy |
| R131 | Hypertrophic Cardiomyopathy (HCM) |
| R132 | Dilated & Arrhythmogenic Cardiomyopathy (DCM & ACM) |
| R133 | Arrhythmogenic Right Ventricular Cardiomyopathy (ARVC) |
| R135 | Paediatric and syndromic cardiomyopathy |

Specific genes tested within each directory reference can be found in the NHS Genomics England Panel App (https://nhsgms-panelapp.genomicsengland.co.uk)

**Supplementary figure 1: Cascade predictive genetic testing and clinical evaluation in relatives**

**Decedent evaluated for inclusion**

**Negative**

**Negative**

**Discharge if adult; ongoing review if child**

**Ongoing management in ICC clinic**

**Relative clinical evaluation**

**Ongoing management in ICC clinic**

**Relative clinical evaluation**

**Discharge**

**Relative predictive genetic test**

**Post-mortem genetic test negative**

**Post-mortem genetic test positive**

**Positive**

**Positive**

**Decedent**

**Relative**

**Supplementary table 3: Decedent demographic data**

| **Decedent data category** | **Data** |
| --- | --- |
| Total decedents included | 107 |
| Age at death (Median) | 35 years (IQR 27.5-45.5) |
| Gender | 67% Male, 33% Female |
| Circumstances of death (top 3) | Rest (45%) Sleep (16%) Exercise (14%) |
| Ethnicity (of reported groups) | White (65%) Black (9%) Asian (7%) |
| Genotype positive (all ages) | 17% |
| Genotype positive ≤ 30 years old | 22% |

**Supplementary table 4: Expected and actual decedent SCD cases included in programme**

| **Site** | **Population size (ages 1-60)** | **(Low) Expected cases over 12-months** | **(High) Expected cases over 12-months** | **Actual cases in peak 12-month period** | **Variance in incident cases** |
| --- | --- | --- | --- | --- | --- |
| **North West** | 512,400 | 8 | 11 | 17 | +6 - +8 |
| **North East** | 561,100 | 9 | 11 | 4 | (5-7) |
| **East** | 855,700 | 13 | 17 | 7 | (6-10) |
| **Central & South** | 941,600 | 15 | 19 | 8 | (7-11) |
| **South West** | 907,800 | 14 | 19 | 11 | (3-8) |
| **North Thames** | 876,800 | 14 | 18 | 1 | (13-17) |
| **South East** | 1,050,900 | 16 | 21 | 15 | (1-6) |
| **Total** | **5,706,300** | **88** | **116** | **63** | **(25 – 53)** |

Based on incidence of 1.55 to 2.04 per 100,000 (Bhatia RT et al^4^)
12-month period selected varied by site and determined by a steady-state period after the site initiated recruitment

**Supplementary table 5: Decedent cases and associated relative genotypes/phenotypes**

| **Decedent case identifier** | **Cause of death** | **Decedent genotype** | **Decedent toxicology findings** | **# Decedent’s relatives completing ICC** | **Decedent’s relatives genotype positive (variant)** | **Decedent’s relatives phenotype positive (diagnosis)** |
| --- | --- | --- | --- | --- | --- | --- |
| 0001 | 1a. Sudden arrhythmic/adult death syndrome (SADS) | Negative | Negative | 6 | 0 | Valvular disease **(2)** |
| 0002 | 2c. Arrhythmogenic cardiomyopathy (ACM)/arrhythmogenic right ventricular cardiomyopathy | Not tested | Negative | 0 | 0 | 0 |
| 0003 | 2b. Dilated cardiomyopathy (DCM) | Negative | Negative | 0 | 0 | 0 |
| 0004 | 1a. Sudden arrhythmic/adult death syndrome (SADS) | Negative | Negative | 0 | 0 | 0 |
| 0005 | 1a. Sudden arrhythmic/adult death syndrome (SADS) | Not tested | Negative | 0 | 0 | 0 |
| 0006 | 5. Other - specify | Negative | Negative | 0 | 0 | 0 |
| 0007 | 4. Thoracic aortic aneurysm +/- dissection / rupture | Negative | Negative | 1 | 0 | 0 |
| 0008 | 2a. Hypertrophic cardiomyopathy (HCM) | MYBPC3:c.2096delC p.(Pro699GlnfsTer55) | Negative | 4 | MYBPC3:c.2096delC p.(Pro699GlnfsTer55) **(3)** | HCM **(1)** |
| 0009 | 1a. Sudden arrhythmic/adult death syndrome (SADS) | Negative | Negative | 1 | 0 | 0 |
| 0010 | 1a. Sudden arrhythmic/adult death syndrome (SADS) | Negative |  | 1 | 0 | 0 |
| 0011 | 1a. Sudden arrhythmic/adult death syndrome (SADS) | Negative | Negative | 0 | 0 | 0 |
| 0012 | 1a. Sudden arrhythmic/adult death syndrome (SADS) | Negative | Negative | 1 | 0 | 0 |
| 0013 | 1a. Sudden arrhythmic/adult death syndrome (SADS) | RYR2:c.1259G>A p.(Arg420Gln) | Not tested | 20 | RYR2:c.1259G>A p.(Arg420Gln) **(4)** | CPVT **(3)** |
| 0014 | 1a. Sudden arrhythmic/adult death syndrome (SADS) | Negative | Negative | 0 | 0 | 0 |
| 0015 | 4. Thoracic aortic aneurysm +/- dissection / rupture | Negative | Negative | 0 | 0 | 0 |
| 0016 | 3. Severe mitral valve prolapse with myxomatous degenerative valvular disease | MYBPC3:c.1224-19G>A | Negative | 12 | MYBPC3:c.1224-19G>A **(6)** | Ventricular tachycardia **(1)** |
| 0017 | 2c. Arrhythmogenic cardiomyopathy (ACM)/arrhythmogenic right ventricular cardiomyopathy | Negative | Negative | 5 | 0 | 0 |
| 0018 | 1a. Sudden arrhythmic/adult death syndrome (SADS) | Negative | Negative | 0 | 0 | 0 |
| 0019 | 5. Other - specify | Negative | Negative | 0 | 0 | 0 |
| 1001 | 5. Other - specify | Negative | Negative | 5 | 0 | 0 |
| 1002 | 2c. Arrhythmogenic cardiomyopathy (ACM)/arrhythmogenic right ventricular cardiomyopathy | Negative | Negative | 4 | 0 | 0 |
| 1003 | 5. Other - specify | Negative | Positive (Synthetic opioid) | 4 | 0 | Borderline CM **(1)** DCM **(1)** |
| 1004 | 2a. Hypertrophic cardiomyopathy (HCM) | Negative | Negative | 2 | 0 | HCM **(1)** |
| 1005 | 4. Thoracic aortic aneurysm +/- dissection / rupture | Negative | Negative | 4 | 0 | 0 |
| 1006 | 2b. Dilated cardiomyopathy (DCM) | Not tested | Positive (Ethanol) | 2 | 0 | Brugada syndrome **(1)** |
| 1007 | 2a. Hypertrophic cardiomyopathy (HCM) | Negative | Positive (2,4 Dinitrophenol) | 4 | 0 | 0 |
| 1008 | 2c. Arrhythmogenic cardiomyopathy (ACM)/arrhythmogenic right ventricular cardiomyopathy | Negative | Negative | 5 | 0 | ACM **(1)** Borderline CM **(1)** |
| 1009 | 2c. Arrhythmogenic cardiomyopathy (ACM)/arrhythmogenic right ventricular cardiomyopathy | DSP:c.3805C>T p.(Arg1269*),   AND   JUP:c.2039G>A p.(Trp680*) | Positive (Promethazine) | 4 | DSP:c.3805C>T p.(Arg1269*) **(1)**;JUP:c.2039G>A p.(Trp680*) **(1);** DSP:c.3805C>T p.(Arg1269*)**AND** JUP:c.2039G>A p.(Trp680*) **(1)** | Borderline CM **(1)** (has the dual mutation) |
| 1010 | 2b. Dilated cardiomyopathy (DCM) | Negative | Negative | 3 | 0 | 0 |
| 1011 | 4. Thoracic aortic aneurysm +/- dissection / rupture | COAL3A1:c.528+5G>A | Negative | 6 | COAL3A1:c.528+5G>A **(3)** | 0 |
| 1012 | 1a. Sudden arrhythmic/adult death syndrome (SADS) | Negative | Negative | 1 | 0 | 0 |
| 1013 | 2c. Arrhythmogenic cardiomyopathy (ACM)/arrhythmogenic right ventricular cardiomyopathy | Negative | Negative | 0 | 0 | 0 |
| 1014 | 2b. Dilated cardiomyopathy (DCM) | Negative | Negative | 0 | 0 | 0 |
| 1015 | 2a. Hypertrophic cardiomyopathy (HCM) | MYBPC3:c.1504C>T (pArg502Trp) | Negative | 2 | MYBPC3:c.1504C>T (pArg502Trp) **(1)** MYBPC3:c.1465G>T p.(Asp489Tyr) **(1)** | 0 |
| 1016 | 2a. Hypertrophic cardiomyopathy (HCM) | Negative | Negative | 1 | 0 | 0 |
| 2001 | 1a. Sudden arrhythmic/adult death syndrome (SADS) | SCN5A:c.5000T>A p.(Val1667Asp) | Negative | 5 | SCN5A:c.5000T>A p.(Val1667Asp) **(1)** | 0 |
| 2002 | 1a. Sudden arrhythmic/adult death syndrome (SADS) | Insufficient sample | Negative | 3 | 0 | Brugada syndrome **(1)** |
| 2003 | 2d. Unexplained cardiac hypertrophy | Negative | Negative | 4 | 0 | Cardiomyopathy **(1)**; DCM **(2)** |
| 2004 | 1a. Sudden arrhythmic/adult death syndrome (SADS) | Negative | Negative | 3 | 0 | Atrial flutter **(1)**; MV prolapse **(1)** |
| 2005 | 1b. Unascertained: Equivocal/uncertain/borderline findings | Negative | Positive (Cannabinoids) | 0 | 0 | 0 |
| 2006 | 1a. Sudden arrhythmic/adult death syndrome (SADS) | Negative | Positive (Sertraline) | 7 | 0 | Borderline CM (1) |
| 2007 | 5. Other - specify | No result available | Negative | 0 | 0 | 0 |
| 2008 | 2d. Unexplained cardiac hypertrophy | Negative | Positive (Ethanol) | 2 | 0 | 0 |
| 2009 | 2c. Arrhythmogenic cardiomyopathy (ACM)/arrhythmogenic right ventricular cardiomyopathy | No result available | Positive (Codeine) | 0 | 0 | 0 |
| 2010 | 2c. Arrhythmogenic cardiomyopathy (ACM)/arrhythmogenic right ventricular cardiomyopathy | Negative | Negative | 0 | 0 | 0 |
| 2011 | 2d. Unexplained cardiac hypertrophy | MYH7:c.732+1G>A | Negative | 1 | MYH7:c.732+1G>A **(1)** | HCM **(1)** |
| 2012 | 4. Thoracic aortic aneurysm +/- dissection / rupture | Negative | Negative | 1 | 0 | Aortic anomaly **(1)** |
| 2013 | 2a. Hypertrophic cardiomyopathy (HCM) | Negative | Negative | 2 | 0 | 0 |
| 2014 | 1a. Sudden arrhythmic/adult death syndrome (SADS) | Negative | Negative | 3 | 0 | 0 |
| 2015 | 2d. Unexplained cardiac hypertrophy | Insufficient sample | Negative | 2 | 0 | Atrial fibrillation **(1)**; LBBB **(1)** |
| 2016 | 2c. Arrhythmogenic cardiomyopathy (ACM)/arrhythmogenic right ventricular cardiomyopathy | Insufficient sample | Negative | 3 | 0 | 0 |
| 2017 | 2d. Unexplained cardiac hypertrophy | Not tested | Not recorded | 0 | 0 | 0 |
| 2018 | 2c. Arrhythmogenic cardiomyopathy (ACM)/arrhythmogenic right ventricular cardiomyopathy | Negative | Negative | 2 | 0 | DCM **(1)** |
| 2019 | 2d. Unexplained cardiac hypertrophy | Negative | Negative | 1 | 0 | 0 |
| 2020 | 3. Severe mitral valve prolapse with myxomatous degenerative valvular disease | Negative | Not recorded | 4 | 0 | MV disease **(1)** |
| 2021 | 4. Thoracic aortic aneurysm +/- dissection / rupture | Negative | Not recorded | 4 | 0 | 0 |
| 2022 | 1b. Unascertained: Equivocal/uncertain/borderline findings | Not tested | Not recorded | 1 | 0 | 0 |
| 2023 | 2c. Arrhythmogenic cardiomyopathy (ACM)/arrhythmogenic right ventricular cardiomyopathy | DSPc.5232dup p.(Lys1745) (2) | Negative | 5 | DSPc.5232dup p.(Lys1745) **(2)** | 0 |
| 2024 | 1a. Sudden arrhythmic/adult death syndrome (SADS) | Negative | Negative | 2 | 0 | 0 |
| 3001 | 1a. Sudden arrhythmic/adult death syndrome (SADS) | Negative | Negative | 0 | 0 | 0 |
| 3002 | 1a. Sudden arrhythmic/adult death syndrome (SADS) | Negative | Not recorded | 3 | 0 | 0 |
| 3003 | 1a. Sudden arrhythmic/adult death syndrome (SADS) | Negative | Not recorded | 0 | 0 | 0 |
| 3004 | 1a. Unascertained: Morphologically normal heart. Sudden arrhythmic/adult death syndrome (SADS) | Pending at end of programme | Not recorded | 0 | 0 | 0 |
| 3005 | 2c. Arrhythmogenic cardiomyopathy (ACM)/arrhythmogenic right ventricular cardiomyopathy | Negative | Negative | 1 | 0 | 0 |
| 3006 | 1a. Sudden arrhythmic/adult death syndrome (SADS) | Negative | Negative | 5 | 0 | 0 |
| 3007 | 5. Other - specify | Pending at end of programme | Not recorded | 0 | 0 | 0 |
| 3008 | 1a. Sudden arrhythmic/adult death syndrome (SADS) | Negative | Negative | 2 | 0 | 0 |
| 4001 | 3. Severe mitral valve prolapse with myxomatous degenerative valvular disease | Negative | Negative | 0 | 0 | 0 |
| 4002 | 2d. Unexplained cardiac hypertrophy | Negative | Negative | 3 | 0 | 0 |
| 4003 | 5. Other - specify | Negative | Negative | 2 | 0 | 0 |
| 4004 | 2e. Unexplained cardiac scarring/fibrosis | KCNE1:c.226G>A p.(Asp76Asn) | Negative | 4 | KCNE1:c.226G>A p.(Asp76Asn) **(1)** | MV prolapse & regurgitation **(1)** |
| 4005 | 1a. Sudden arrhythmic/adult death syndrome (SADS) | Negative | Negative | 4 | 0 | PAF **(1)** |
| 4006 | 1a. Sudden arrhythmic/adult death syndrome (SADS) | Negative | Negative | 2 | 0 | CPVT-CRDS spectrum **(1)** |
| 4007 | 4. Thoracic aortic aneurysm +/- dissection / rupture | Negative | Negative | 3 | 0 | Aortic dilatation **(1)** ASD **(1)** |
| 4008 | 5. Other - specify | Negative | Negative | 3 | 0 | LVNT **(1)** |
| 4009 | 4. Thoracic aortic aneurysm +/- dissection / rupture | Negative | Negative | 1 | 0 | AVR and conduction disease **(1)** |
| 4010 | 4. Thoracic aortic aneurysm +/- dissection / rupture | FBN1:c.1426T>A p.(Cys476Ser) | Negative | 2 | FBN1:c.1426T>A p.(Cys476Ser) **(1)** | Marfan's syndrome **(1)** |
| 4011 | 1a. Sudden arrhythmic/adult death syndrome (SADS) | Negative | Positive (Sertraline) | 3 | 0 | 0 |
| 4012 | 5. Other - specify | Negative | Negative | 2 | 0 | Ischaemic heart disease **(1)** |
| 4013 | 5. Other - specify | Not tested | Positive (Ethanol, cocaine) | 1 | 0 | 0 |
| 5001 | 2a. Hypertrophic cardiomyopathy (HCM) | LAMP2:chrX:120431322-120469169 | Negative | 0 | 0 | 0 |
| 5002 | 1a. Sudden arrhythmic/adult death syndrome (SADS) | Negative | Negative | 1 | 0 | 0 |
| 5003 | 2b. Dilated cardiomyopathy (DCM) | Negative | Positive (Ethanol) | 0 | 0 | 0 |
| 5004 | 1a. Sudden arrhythmic/adult death syndrome (SADS) | Negative | Not tested | 1 | 0 | 0 |
| 5005 | 1a. Sudden arrhythmic/adult death syndrome (SADS) | Negative | Negative | 0 | 0 | 0 |
| 5006 | 2c. Arrhythmogenic cardiomyopathy (ACM)/arrhythmogenic right ventricular cardiomyopathy | Negative | Negative | 1 | 0 | ACM **(1)** |
| 5007 | 1a. Unascertained: Morphologically normal heart. Sudden arrhythmic/adult death syndrome (SADS) | Negative | Negative | 0 | 0 | 0 |
| 5008 | 5. Other - specify | Negative | Negative | 2 | 0 | 0 |
| 5009 | 1a. Sudden arrhythmic/adult death syndrome (SADS) | Negative | Negative | 0 | 0 | 0 |
| 5010 | 5. Other - specify | No result available | Positive (not specified) | 0 | 0 | 0 |
| 5011 | 1a. Sudden arrhythmic/adult death syndrome (SADS) | No result available | Negative | 0 | 0 | 0 |
| 5012 | 3. Severe mitral valve prolapse with myxomatous degenerative valvular disease | Negative | Not recorded | 0 | 0 | 0 |
| 5013 | 2e. Unexplained cardiac scarring/fibrosis | Negative | Negative | 3 | 0 | 0 |
| 5014 | 2c. Arrhythmogenic cardiomyopathy (ACM)/arrhythmogenic right ventricular cardiomyopathy | FLNC:c.4333_4340delins20 p.(Lys1445_Lys1447delinsProGlyGlnTer) | Negative | 0 | 0 | 0 |
| 5015 | 2e. Unexplained cardiac scarring/fibrosis | Negative | Negative | 0 | 0 | 0 |
| 5016 | 2e. Unexplained cardiac scarring/fibrosis | Negative | Negative | 1 | 0 | 0 |
| 5017 | 2b. Dilated cardiomyopathy (DCM) | Negative | Negative | 0 | 0 | 0 |
| 5018 | 2b. Dilated cardiomyopathy (DCM) | Negative | Negative | 1 | 0 | 0 |
| 5019 | 2c. Arrhythmogenic cardiomyopathy (ACM)/arrhythmogenic right ventricular cardiomyopathy | DSP:c.7773_7776del p.(Ser2591ArgfsTer11) | Negative | 0 | 0 | 0 |
| 5020 | 1a. Sudden arrhythmic/adult death syndrome (SADS) | Insufficient sample | Not tested | 0 | 0 | 0 |
| 6001 | 1a. Unascertained: Morphologically normal heart. Sudden arrhythmic/adult death syndrome (SADS) | No result available | Negative | 3 | 0 | 0 |
| 6002 | 1a. Sudden arrhythmic/adult death syndrome (SADS) | Negative | Negative | 4 | 0 | 0 |
| 7001 | 1a. Sudden arrhythmic/adult death syndrome (SADS) | KCHN2:c.2775dup p.(Pro926AlafsTer14) | Negative | 24 | KCHN2:c.2775dup p.(Pro926AlafsTer14) **(13)** | Long QT **(5)**; Palpitations **(1)**; Pre-syncope **(2)**; Syncope **(4)** |
| 7002 | 1a. Sudden arrhythmic/adult death syndrome (SADS) | Negative | Negative | 0 | 0 | 0 |
| 7003 | 2b. Dilated cardiomyopathy (DCM) | Negative | Negative | 0 | 0 | 0 |
| 7004 | 2c. Arrhythmogenic cardiomyopathy(ACM)/arrhythmogenic right ventricular cardiomyopathy | Not tested | Negative | 0 | 0 | 0 |
| 7005 | 2b. Dilated cardiomyopathy (DCM) | Negative | Negative | 0 | 0 | 0 |

Number in brackets indicates the number of the decedent’s relatives to whom the genotype or phenotype applies
