# Supplementary material for: Prospective use of genomics in the evaluation of sudden cardiac death: results from a national health service population pathway
Source: eBioMedicine. 2026 May 13;128:106266. doi: 10.1016/j.ebiom.2026.106266 (PMC13261715; doi:10.1016/j.ebiom.2026.106266)
Supplement: Collab Authors [file mmc2.docx]

*NHS and Coronial Service Sudden Unexpected Death programme group* *members*

| **First Name** | **Last Name** | **Affiliation** |
| --- | --- | --- |
| **NHS ICC Coordinators** | | |
| Georgiana | Neculau | Central & South GMSA, Birmingham |
| Laura | Vilajuana | East GMSA, Leicester |
| Kim | Clark | North West GMSA, Manchester |
| Nina | Edwards | South West GMSA, Bristol |
| Elaine | Hobson | North East & Yorkshire GMSA, Sheffield |
| Claire | Kirby | North Thames GMSA, North London |
| Katie | Frampton | South East GMSA, South London |
| **NHS ICC Clinical Service Leads** | | |
| Graham | Stuart | South West GMSA, Bristol |
| Bode | Ensam | Central & South GMSA, Birmingham |
| Nigel | Wheeldon | North East & Yorkshire GMSA, Sheffield |
| Perry | Elliott | North Thames GMSA, North London |
| William | Newman | North West GMSA, Manchester |
| Harshil | Dhutia | East GMSA, Leicester |
| Greg | Mellor | East GMSA, Cambridge |
| Jan | Till | South East GMSA, South London |
| **British Heart Foundation Programme Team** | | |
| Judy | O'Sullivan | British Heart Foundation |
| Iain | Armstrong | British Heart Foundation |
| Andrew | Leatherland | British Heart Foundation |
| Jennifer | Townsend | British Heart Foundation |
